# Peptide-based vaccine targeting IL17A attenuates experimental spondyloarthritis in HLA-B27 transgenic rats

**DOI:** 10.1136/rmdopen-2022-002851

**Published:** 2023-02-03

**Authors:** Hiroki Hayashi, Jiao Sun, Yuka Yanagida, Shota Yoshida, Satoshi Baba, Akiko Tenma, Masayoshi Toyoura, Sotaro Kawabata, Takako Ehara, Ryoko Asaki, Makoto Sakaguchi, Hideki Tomioka, Munehisa Shimamura, Ryuichi Morishita, Hiromi Rakugi, Tetsuya Tomita, Hironori Nakagami

**Affiliations:** 1 Department of Health Development and Medicine, Osaka University Graduate School of Medicine, Suita, Osaka, Japan; 2 Department of Geriatric and General Medicine, Osaka University Graduate School of Medicine, Suita, Osaka, Japan; 3 FunPep.Co, Suita, Osaka, Japan; 4 Department of Neurology, Osaka University Graduate School of Medicine, Suita, Osaka, Japan; 5 Department of Clinical Gene Therapy, Osaka University Graduate School of Medicine, Suita, Osaka, Japan; 6 Graduate School of Health Science, Morinomiya University of Medical Sciences, Osaka, Osaka, Japan

**Keywords:** Spondylitis, Ankylosing, Vaccination, Autoimmunity

## Abstract

**Objectives:**

Spondyloarthritis (SpA) is known as series of immune-mediated inflammatory disease of the axial and peripheral joints. Human leucocyte antigen (HLA)-B27 is a genetic risk factor for SpA. Recent evidence suggests that the interleukin −17 (IL17) axis strongly contributes to SpA. This study aimed to assess the efficacy of an IL17A peptide-based vaccine on SpA manifestations in model rats.

**Methods:**

HLA-B27/human β_2_-microglobulin (hβ_2_M) transgenic rats were immunised with heat-inactivated *Mycobacterium tuberculosis* (MT) to develop spondylitis and arthritis as an experimental SpA model after immunisation with a keyhole limpet hemocyanin-conjugated IL17A peptide-based vaccine with an alum adjuvant three times. The IL17A antibody titre was assessed using ELISA, and arthritis score and joint thickness were monitored two times a week. Enzyme-linked immunospot (ELISpot) assays for IL4- and interferon-γ-secreting splenocytes were conducted to evaluate IL17A-specific T cell activation. We also evaluated the effect of IL17A vaccine in SpA therapeutic model.

**Results:**

The IL17A peptide-based vaccine with alum adjuvant successfully induced antibody production and suppressed the arthritis score and joint thickness. X-ray and histological analyses showed that enthesitis, bone destruction and new bone formation were inhibited by the IL17A vaccine. The ELISpot assay showed that the IL17A peptide-based vaccine did not elicit any IL17A-reactive T cell responses. IL17A vaccine tends to mitigate, but not significant, in SpA treatment model. These data showed that the peptide-based vaccine targeting IL17A alleviated the SpA phenotype in a heat-inactivated MT-induced SpA model in HLA-B27/hβ_2_M transgenic rats.

**Conclusions:**

IL17A peptide-based vaccine may be a therapeutic option for SpA treatment.

**What is already known about this subject?**

- Interleukin-17A (IL17A) is known to have a pivotal role in inflammation and bone metabolism in spondyloarthritis (SpA). Antibody therapy against IL17A is effective but expensive in the clinical setting.

**What does this study add?**

- IL17A vaccine has a beneficial effect on SpA model rats without induction of auto-reactive T cell against IL17A.

**How might this impact on clinical practice?**

- IL17A vaccine might be an additional therapeutic approach for SpA treatment.

## Introduction

Spondyloarthritis (SpA), including ankylosing spondylitis, is triggered at the tendons and enthesis in sacroiliac joints of the spine via an immune-mediated inflammatory mechanism, resulting in ankylosis with bone erosion and new bone formation.^1^ Many studies suggested that major histocompatibility complex class I antigen B27 (human leucocyte antigen; HLA-B27) is associated with axial SpA or ankylosing spondylitis.^2–8^ Transgenic rats with HLA-B27 and human β_2_-microglobulin (hβ_2_M) have been established and analysed to verify the relationship between HLA-B27 and the manifestations of SpA, including inflammation in peripheral and axial joints and other tissues.^9 10^ To date, the precise mechanism(s) of articular inflammation leading to SpA remains unknown.

In addition to HLA-B27, recent evidence suggests that the interleukin (IL)−23/IL-17 (IL23/IL17) axis strongly contributes to the pathological mechanism that triggers SpA.^11–13^ IL17A is involved in arthritogenic inflammation via the regulation of Wnt and receptor activator of nuclear factor-κB ligand pathways and new bone formation via the Janus kinase/signal transducer and activator of transcription pathway at the joints and entheses.^14^ Based on these studies, some biological therapies using neutralising antibodies targeting IL17A, such as secukinumab (a fully human monoclonal antibody) and ixekizumab (a humanised monoclonal antibody), have been evaluated in clinical studies.^15–18^

As the efficacy of these biological antibodies has been approved, more therapeutic options are available for SpA treatment. However, biological therapy is expensive and burdensome for patients, even with medical insurance support.^19^ To address this issue, we have focused on a therapeutic vaccine through which individual therapeutic antibodies can be produced.^20^ We have previously demonstrated the effectiveness of potential therapeutic vaccines for hypertension, diabetes mellitus and stroke.^21–25^ In this study, we investigated the efficacy of an IL17 vaccine against SpA manifestations in HLA-B27/hβ_2_M transgenic rats.

In this study, we developed a peptide-based vaccine for IL17A based on our previous findings and evaluated its efficacy against the manifestations of SpA in a rat model.

## Methods

### HLA-B27 transgenic rats, induction of SpA-like phenotype and scoring of arthritis

Lewis HLA-B27/hβ_2_M transgenic rats were obtained from the University of Texas Southwestern Medical Center by crossing two transgenic strains, as previously described.^9^ Namely, the 21–3 (male) and 283–2 (female) transgenic rats were crossed, and the tails of their littermates were analysed for transgenes via genotyping. All rats were maintained with light/dark cycles every 12 hours. All animal experiments were approved by the Animal Experiment Committee of Osaka University.

To induce the spondylitis and arthritis phenotype, heat-inactivated *Mycobacterium tuberculosis* (MT) (90 µg) mixed with the incomplete Freund’s adjuvant (total amount: 100 µL) was subcutaneously injected 3 weeks after the first IL17A vaccination.^10^ After MT injection, the body weight, joint thickness and spondylarthritis severity were monitored every 2–3 days. The paw joint thickness was measured using an electric calliper. The arthritis score was calculated based on the swelling scores of each finger and tail (none=0; mild swelling=1; severe swelling=2). MT-treated HLA-B27 tg rats were used as positive control (N=5). As negative control, HLA-B27-negative rats were used (N=1–2). For SpA treatment model, MT was administrated, followed by IL17A vaccine injection three times at 2 weeks interval. MT-treated HLA-B27 tg rats were used as positive control (N=6). As negative control, HLA-B27 negative rats were used (N=2).

### Peptide preparation and immunisation

The IL17A epitope peptide was synthesised by the Peptide Institute (Osaka, Japan). The amino acid sequences from 65aa to 72aa were selected as epitopes for IL17A.^24^ The synthesised peptide was conjugated with keyhole limpet hemocyanin (KLH; Enzo Life Sciences) as a carrier protein and mixed with an aluminium adjuvant (InVivoGen). The KLH-conjugated vaccine or KLH-only as a control (100 µg/body) was subcutaneously injected into the rats three times at 0, 2 and 4 weeks (N=4–6).

### Antibody titre determination via ELISA

Serum samples from the tail vein at 0, 2, 4, 6 and 9 weeks were evaluated via ELISA for antibody titres, as described in a previous study. Briefly, bovine serum albumin (Peptide Institute)-conjugated epitope (10 µg/mL) or recombinant IL17A (R&D Systems) was coated on a 96-well plate on the first day. On the second day, the wells were blocked with the blocking buffer (PBS-T (Tween-20, 0.05%) containing 5% skim milk) for 2 hours at room temperature. The sera were diluted 10-fold to 31 250-fold in a blocking buffer and incubated overnight at 4℃. The following day, the wells were washed and incubated with horseradish peroxidase (HRP)-conjugated anti-IgG antibody (GE Healthcare) for 3 hours at room temperature. HRP-conjugated antisubclass IgG antibodies (IgG1: Abcam; IgG2a: Abcam) were used for IgG subclass analysis. After washing with PBS-T, the wells were incubated with the peroxidase chromogenic substrate, 3,3’−5,5’-tetramethyl benzidine (Sigma-Aldrich), for 30 min at room temperature, and the reaction was halted with 0.5 N sulfuric acid. The absorbance of the wells was measured immediately at 450 nm using a microplate reader (Bio-Rad). The antibody titre was shown as the serum dilution with half-maximal binding (optical density: 50%). The half-maximal antibody titre of each sample was calculated from the highest absorbance in the dilution range using the Prism V.8 software.

### Enzyme-Linked immunospot (ELISpot) assay

Cellular immune responses of splenocytes after vaccination were evaluated via an ELISpot assay (UCT Biosciences), according to the manufacturer’s instructions. The polyvinylidene fluoride membrane-coated plates were incubated with IL4 or interferon (IFN)γ capture antibodies at 4℃ overnight. The wells were washed with PBS and blocked with the blocking solution (UCT Biosciences) for 2 hours at room temperature. Splenocytes were isolated from vaccinated spleens and adjusted to 2×10^5^ cells/well. The cells were stimulated with recombinant IL17A (R&D Systems), KLH (Enzo Life Sciences) or phorbol 12-myristate 13-acetate (PMA; Sigma) plus ionomycin (IO; Sigma) at 37 ℃ for 48 hours. The washed wells were incubated with a biotinylated polyclonal antibody against rat IL4 or IFNγ for 2 hours at 4℃. After the wells were washed with PBS-T, they were incubated with the streptavidin-HRP conjugate for 1 hour at room temperature. HRP was developed using a substrate solution (UCT Biosciences). IL4 or IFNγ spots were counted using a dissecting microscope (LMD6500; Leica).

### Histological analysis

For histological analysis, tissues (joint, tail, kidney, liver and lungs) were collected 9 weeks after the first vaccination and fixed in 10% neutral buffered formalin. Fixed tissues were embedded in paraffin, cut into 5 µm-thick sections and stained with H&E.

For immunohistological analysis, paraffin-embedded tissues were cut into 3 µm-thick sections and stained with CD68 (Bio-Rad), CD14 (Bioss antibodies) and alkaline phosphatase (ALP; Santa Cruz). Stained tissue sections were observed under a BZ-X810 microscope (Keyence).

### Radiologic analysis (X-ray analysis)

Rats were imaged with X-rays at the end of the experiments using the MX-20 specimen radiography system, as previously described.^26^

### Statistical analysis

All values are presented as the mean±SE of the mean. Student’s t test and one-way analysis of variance followed by Tukey’s post hoc multiple test were used to assess the significant differences in each experiment using Prism V.8 software (GraphPad Software). Differences were considered significant when the p value was less than 0.05.

## Results

### Peptide-based IL17A vaccine induces IL17A-specific antibody production in HLA-B27/hβ_2_M transgenic rats

**Figure 1:**
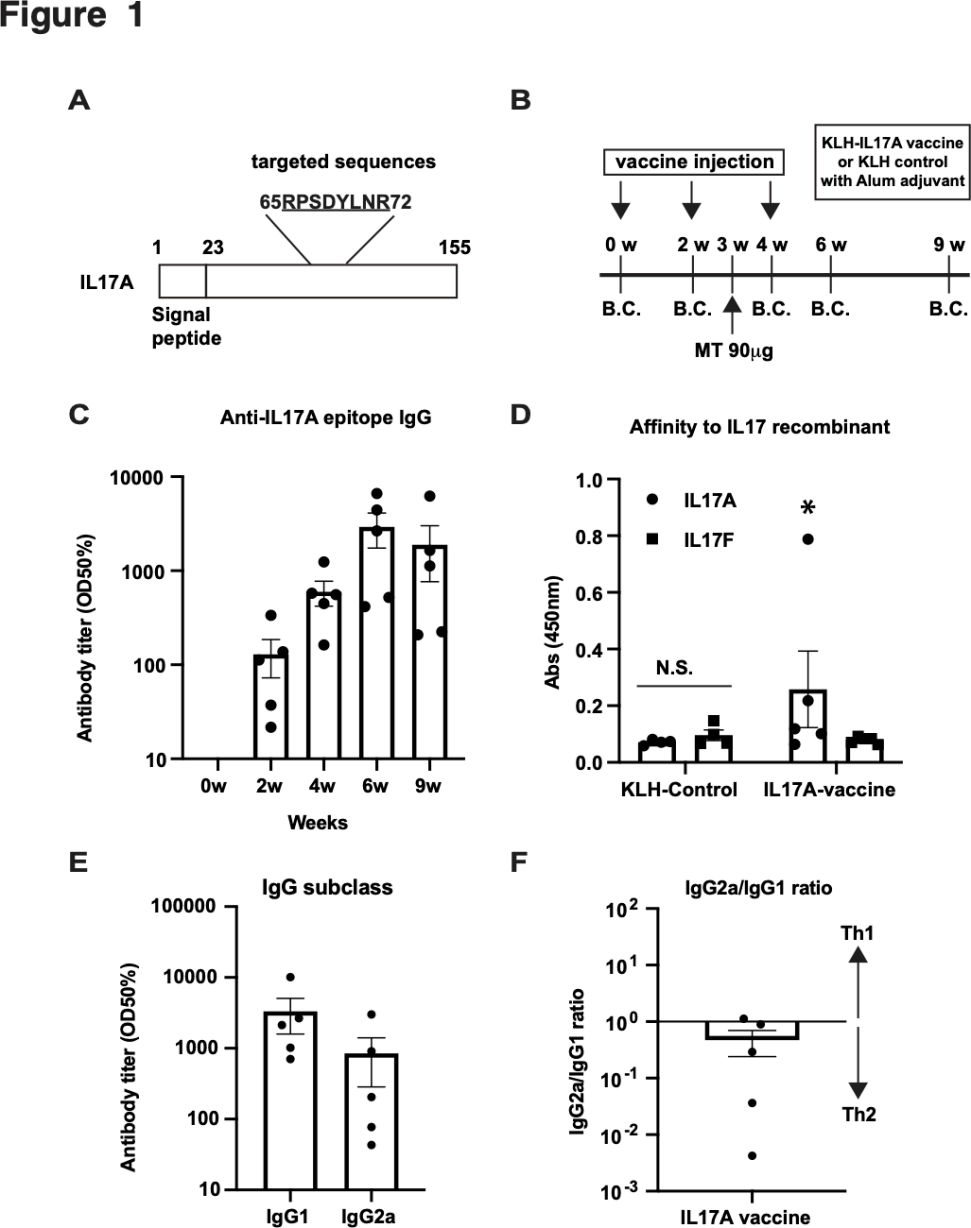
Interleukin (IL)−17A (IL17A) vaccine induces antibody production in a spondyloarthritis (SpA) model in human leucocyte antigen (HLA)-B27 transgenic rats. (A) Targeted sequences of IL17A vaccine are shown. (B) Experimental protocol for IL17A vaccination of the inactivated *Mycobacterium Tuberculosis* (MT)-induced SpA model in HLA-B27 transgenic rats. The epitope was conjugated with keyhole limpet hemocyanin (KLH). KLH-conjugated IL17A vaccine was injected with an alum adjuvant. HLA-B27 transgenic rats were immunised with the IL17A vaccine with KLH or KLH only intracutaneously thrice every 2 weeks. Inactivated MT was administrated to induced SpA. Blood was collected at 0, 2,4, 6 and 9 weeks to evaluate the antibody titre. Joint, tail and other tissues were collected at 9 weeks. The arthritis score and joint thickness were evaluated every few days. B.C.: blood collection. (C) Antibody titre for IL17A epitope. (D) Binding affinity of the vaccine-induced antibody for recombinant IL17A analysed via ELISA. Antibody of immunised serum at 9 weeks showed specific binding to recombinant IL17A, but not recombinant IL17F. (E) Antibody titre of IL17A epitope-specific IgG subclasses, IgG1 (Th2) and IgG2a (Th1). (F) The balance of Th1/Th2 was evaluated by the IgG2a/IgG1 ratio. IgG2a antibody titre was individually divided by the IgG1 antibody titre. See also online supplemental figure 1.

IL17A functions as a homodimer or heterodimer with IL17A or IL17F.^27^ The interface for the dimer, which is an amino acid sequence, was chosen as the epitope for the vaccine in this study, which consists of amino acid sequences, RPSDYLNR from 65aa to 72aa (figure 1A). This epitope was conjugated at the N-terminus with KLH as a carrier protein. The KLH-conjugated vaccine (100 µg peptide/rat) in combination with an alum adjuvant was subcutaneously injected into rats three times every 2 weeks (figure 1B). Heat-inactivated MT (90 µg/rat) was administered to rats to induce SpA 3 weeks after the first vaccine^23^ (figure 1B). The antibody titre was measured at 0, 2, 4, 6 and 9 weeks using ELISA. The antibody titre increased from week 2 to its highest value at week 6 (figure 1C and online supplemental figure 1). The antibody level was not increased by the KLH-control vaccine. Next, we examined whether the IL17A vaccine-induced antibodies recognised and bound to recombinant IL17A using ELISA. The IL17A vaccine-induced antibody at 9 weeks recognised recombinant IL17A but not IL17F (figure 1D). To assess the Th1:Th2 balance induced by the alum-adjuvanted IL17A vaccine, IgG subclass analysis was performed using ELISA. The IgG1 antibody titre was found to be dominant (figure 1E) compared with the IgG2a antibody titre. The IgG2a/IgG1 ratio showed that the IL17A vaccine with alum adjuvant shifted the IL17A immune response towards Th2 (figure 1F). These data suggest that the IL17A vaccine successfully induces Th2-shifted IL17A-specific antibody production in the SpA model in HLA-B27/hβ_2_M transgenic rats.

10.1136/rmdopen-2022-002851.supp1 Supplementary data

### Manifestations of SpA are significantly alleviated by the peptide-based IL17A vaccine in HLA-B27/hβ_2_M transgenic rats

**Figure 2:**
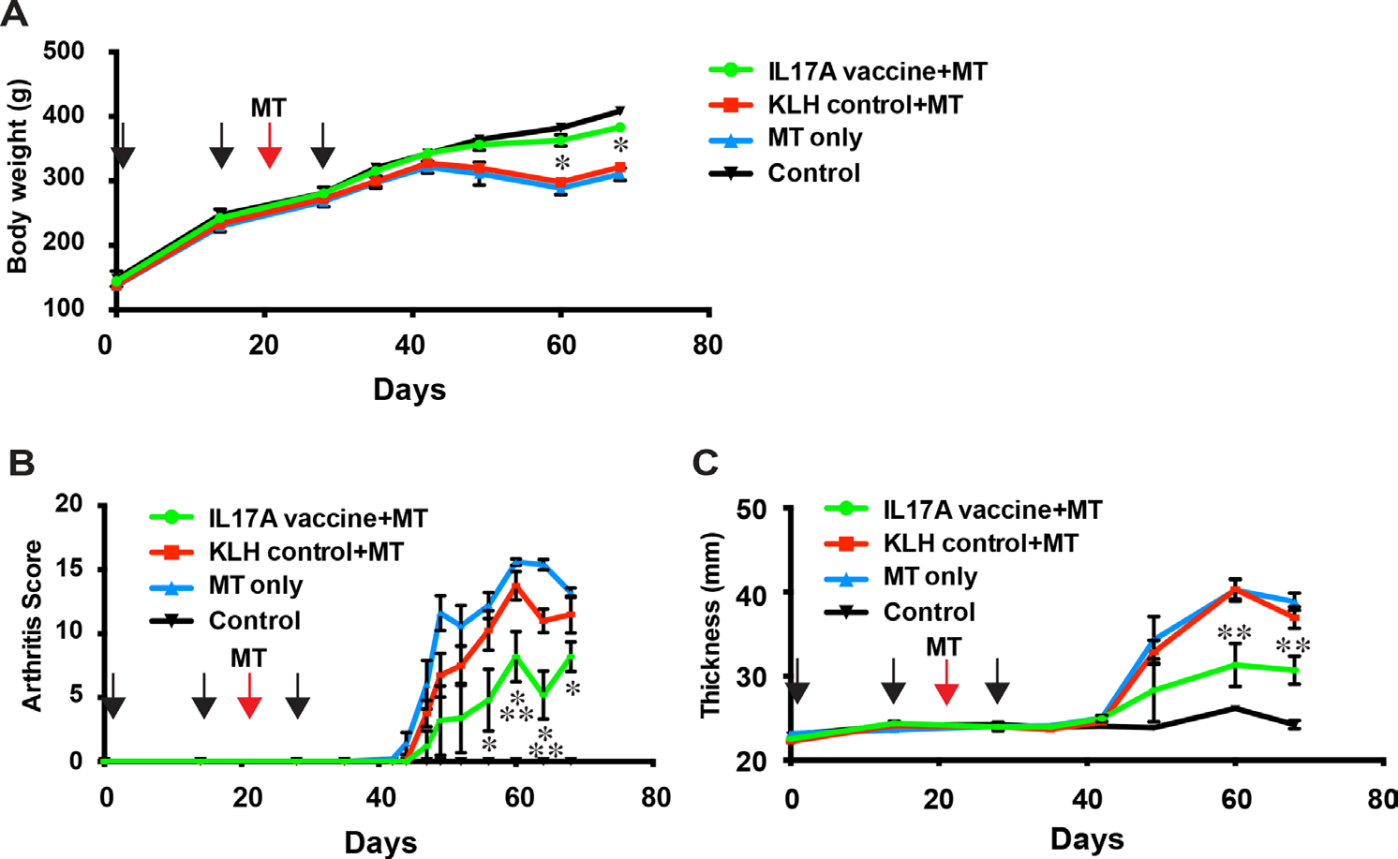
IL17A vaccine alleviates Mycobacterium tuberculosis (MT)-induced spondyloarthritis (SpA) in HLA-B27 transgenic rats. (A) Body weight was monitored after IL17A vaccination. (B) Arthritis score and (C) joint thickness were analysed every 2–3 d to determine the arthritis severity. Data are shown as the mean ± SE error of the mean (SEM). *p < 0.05 versus MT only, **p < 0.05 versus MT only, and KLH-control+MT, respectively.

To test the effect of the IL17A vaccine on SpA, we evaluated the arthritis score and joint thickness of rats administered IL17A or KLH-control vaccine every few days after MT injection. We monitored the body weight and clinical scoring at the joints and tail macroscopically. Bodyweight decreased in the KLH control+MT vaccine and MT-only groups (figure 2A). We confirmed that arthritis severity and swelling at the joint developed around 20 days after MT immunisation in the control-KLH vaccine (KLH control+MT) and no-treatment (MT only) groups. The severity of arthritis score and joint swelling induced by MT immunisation was significantly alleviated (figure 2B, C). These results suggest that the IL17A vaccine successfully attenuates the SpA phenotype.

### IL17A vaccine reduces the axial and peripheral inflammation in HLA-B27 transgenic rats

**Figure 3:**
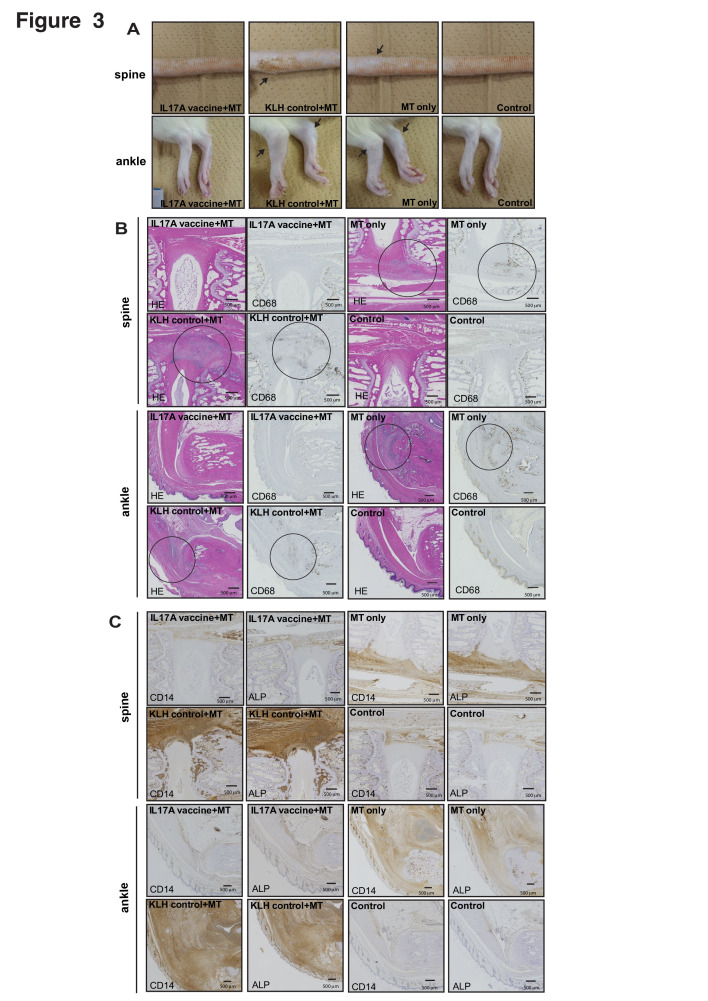
IL17A vaccine suppresses enthesitis and bone remodelling in the joints of HLA-B27 transgenic rats. (A) Representative pictures of spine and ankle. Arrows indicate swelling. (B) Immunohistological analysis (HE and CD68 staining) of the joint of spine and ankle. Circle indicates invasion of immune cells. (C) Immunohistological analysis (CD14 and alkaline phosphatase (ALP) staining) of the joint of spine and ankle at 9 weeks. See also online supplemental figure 2 and 3.

Inhibition of SpA-related manifestations in the spine joints, as shown in figure 2, suggests that the IL17A vaccine suppresses inflammation. Six weeks after MT immunisation, swelling of axial (spine) and peripheral (ankle) joints was observed in the KLH-control (KLH control+MT) and no treatment (MT only) groups but to a lesser extent in the IL17A vaccine group (figure 3A). Bone destruction and bone formation were detected via X-ray analysis in ankle joint but not in spine (online supplemental figure 2). Next, we stained the spinal and peripheral joint sections with HE staining as well as markers of inflammation (CD68) and bone formation (CD14 and ALP). We detected immune cell invasion and CD68 staining in the KLH-control (KLH control+MT) and no treatment (MT only) groups. However, the IL17A vaccine suppressed cell invasion and CD68-positive inflammatory cells during enthesis (figure 3B and online supplemental figure 3A). We also evaluated bone formation via CD14 and ALP staining. Similar to the results for inflammatory markers, CD14-positive and ALP-positive cells were increased in the KLH-control (KLH control+MT) and the no-treatment (MT only) groups compared with the IL17A vaccine (IL17A vaccine+MT) and control groups (figure 3C and online supplemental figure 3B). These results suggest that the IL17A vaccine suppresses enthesitis and bone destruction/formation in the joints of HLA-B27 transgenic rats.

### IL17A peptide-based vaccine does not induce IL17A-specific T cell activation

**Figure 4:**
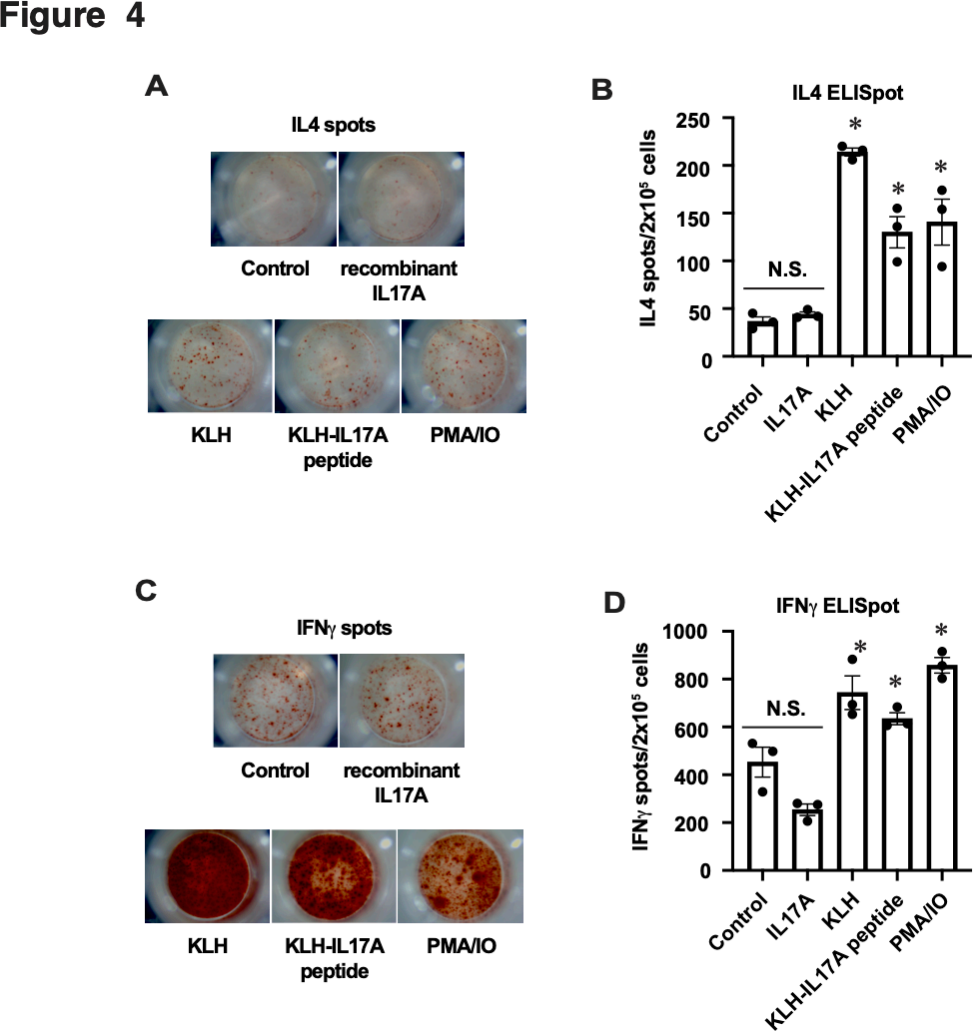
IL17A peptide vaccine does not activate IL17A-specific T cell reaction. Representative images of (A) IL4 or (C) interferon (IFN)γ spots obtained via the enzyme-linked immunospot (ELISpot) assay. Splenocytes from immunised HLA-B27 transgenic rats were stimulated with recombinant IL17A, KLH or KLH-IL17 peptide (vaccine), or phorbol 12-myristate 13-acetate (PMA) plus ionomycin (IO) (PMA/IO) as a positive control. The graph shows the number of (B) IL4 or (D) IFNγ spots per 2 x 10^5^ cells. Data are shown as the mean ± SEM. *p < 0.05 versus Control and IL17A, respectively.

Next, we evaluated the safety of the IL17A vaccine. Vaccine-elicited autoimmune T cell reactions to endogenous IL17A protein pose a major safety concern. To assess this, the cellular immune response was evaluated via an ELISpot assay using immunised splenocytes. KLH stimulation increased the number of IL4 spots compared with IL17A stimulation or the control. IL4 spots after IL17A stimulation were comparable to those without stimulation (control) (figure 4A, B). The number of IFNγ-producing splenocytes increased with KLH stimulation but not with IL17A stimulation (figure 4C, D), suggesting that the IL17A-autoreactive T cells were not produced by the IL17A vaccine in this study.

### IL17A peptide vaccine tend to have therapeutic effect in MT-induced SpA treatment model, but not significant

**Figure 5:**
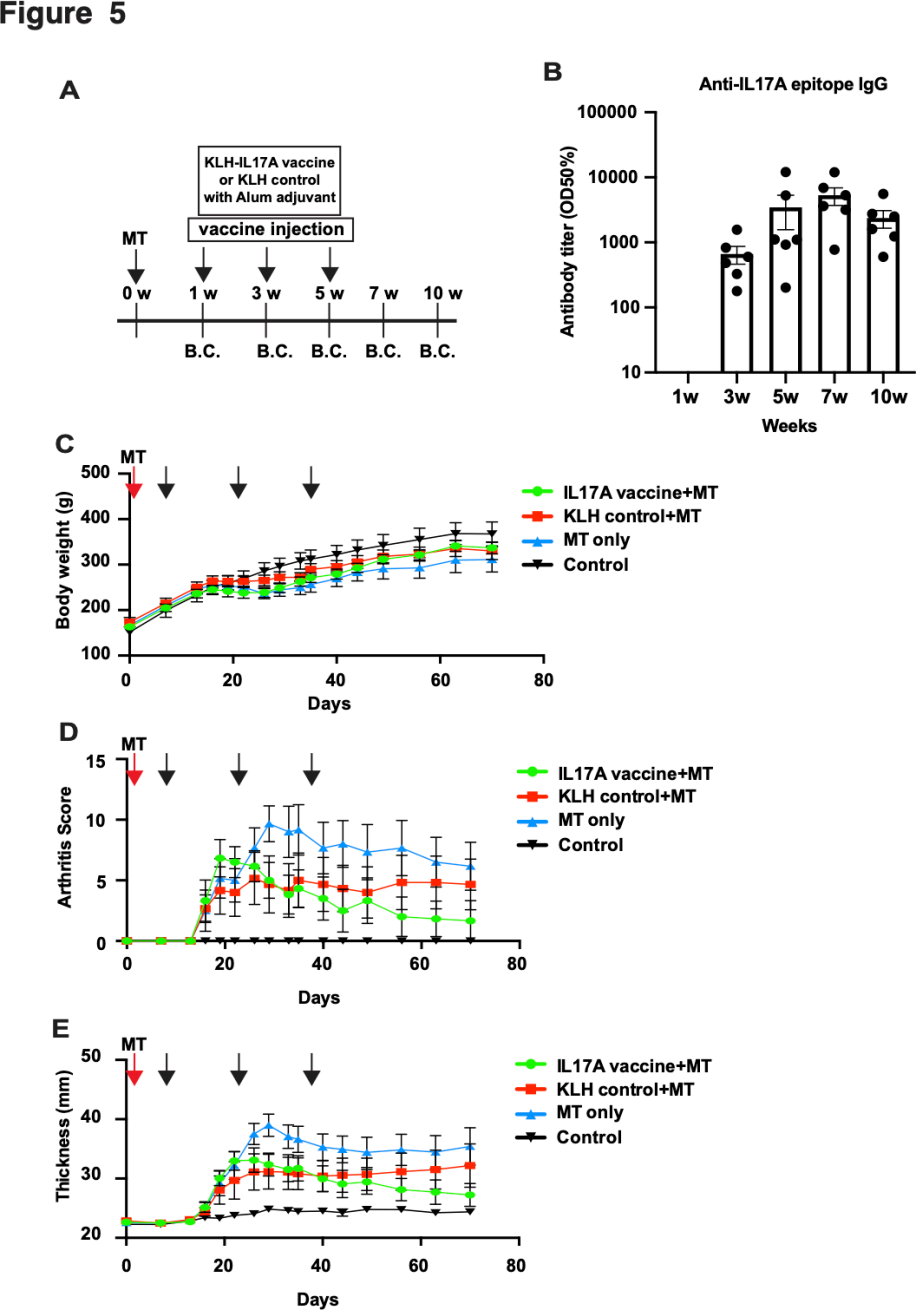
IL17A peptide vaccine does not mitigate MT-induced SpA in treatment model. (A) Experimental protocol for IL17A vaccination of MT-induced SpA treatment model in HLA-B27 transgenic rats. MT was administered to induce SpA at a week before administration of IL17A vaccine. IL17A vaccine or KLH control was administrated thrice at 2 weeks interval. Blood was collected at 1, 3, 5, 7 and 10 weeks to evaluate the antibody titre. The arthritis score and joint thickness were evaluated every few days. B.C.: blood collection. (B) Antibody titre for IL17A epitope. (C) Body weight was monitored after MT administration. (D) Arthritis score and (E) joint thickness were analysed every 2–3 days to determine the arthritis severity. Data are shown as the mean ± SE error of the mean (SEM). See also online supplemental figure 4.

Finally, we evaluated whether IL17A vaccine has a beneficial effect on MT-induced SpA in therapeutic model. HLA-B27 transgenic rats were immunised with MT before IL17A vaccine administration,^28^ followed by three doses of IL17A vaccine at 2-week interval. We evaluated the arthritis score and joint thickness every few days (figure 5A). IL17A epitope-specific antibody titre was increased, measured by ELISA (figure 5B and online supplemental figure 4). Body weight was not significantly different among groups (figure 5C). Arthritis score and joint thickness were not significantly different in time window in this study (figure 5D, E). However, arthritis score and joint thickness tend to relieve from 40 days to 44 days since MT injection in IL17A vaccine group compared with KLH control group. Collectively, IL17A vaccine did not show the therapeutic effect in 10 weeks in therapeutic model.

## Discussion

In the present study, we showed that the IL17A vaccine successfully induced antibody production by specifically binding to IL17A but not IL17F. The arthritis score and thickness of the hind limb paw were strongly suppressed after the SpA-like phenotype was induced by heat-inactivated MT. Histological analysis revealed that articular inflammation and new bone formation were inhibited at the joints and spine. Moreover, the IL17A vaccine did not cause IL17A-specific T cell activation as well as any tissue damage in the lungs, kidneys and liver. These data suggest that therapeutic vaccines targeting IL17A protect against SpA manifestations in HLA-B27/β_2_M transgenic rats, without causing any adverse effects.

IL17A vaccine has been used to treat IL17A-related autoimmune disorders.^29–32^ IL17A vaccine was first developed to prevent experimental autoimmune encephalomyelitis (EAE) in an animal model in 2006. In that study, mice immunised with recombinant ovalbumin-conjugated mouse IL17A with Gerbu 100 adjuvant produced an IL17A-specific antibody, which suppressed IL17A bioactivity. The IL17A vaccine successfully inhibited the symptoms of proteolipid protein peptide 139–151-induced EAE in Swiss Jim Lambert (SJL) mice.^32^ IL17A peptide-based vaccine has also been used to treat psoriasis. That study revealed that immunisation with the Q11 self-assembling nanofiber system using multiple IL17A peptides and the universal T-cell epitope, Pan DR T-Helper Epitope (PADRE), increased the antibody titre in mice. Alum-adjuvanted Q11-IL17A vaccine prevents psoriasis in imiquimod-induced model mice by shifting antibody production towards IgG1 vs IgG2b.^31^ In addition to the peptide-based vaccine, our previous study showed the beneficial effects of the IL17A DNA vaccine for systemic lupus erythematosus (SLE). A DNA vaccine encoding a hepatitis B core (HBc)-IL17A epitope fusion protein successfully induced anti-IL17A IgG production via intramuscular administration with electroporation and protected the SLE model mice (NZBWF1 or MRL/lpr mice) from pathological symptoms, such as macrophage infiltration and renal damage.^30^ Virus-like particle-based IL17A vaccine also induces IL17A antibodies, which worsens chronic colitis in a 2,4,6-trinitrobenzene sulfonic acid-induced colitis model in mice.^29^ Indeed, basic and clinical studies using IL17A targeting monoclonal antibodies to treat inflammatory bowel disease (IBD), such as Crohn’s disease, showed unfavourable results.^33–36^ This suggests that the IL17A vaccine may be applicable for IL17A-related pathologies, such as SpA, psoriasis, EAE, and SLE but not IBD.

In this study, the IL17A epitope peptide-based vaccine-suppressed SpA manifestations in SpA model rats without any toxic effects, such as body weight loss (figure 2A). Our previous study using DNA vaccine encoding the same IL17A epitope, RPSDYLNR, showed that the splenocytes from immunised mice were not activated by the IL17A epitope or recombinant IL17A, suggesting that the IL17A vaccine does not induce autoreactive T cells.^30^ In our previous studies, KLH-conjugated vaccines targeting endogenous molecules did not induce autoimmune T cells^21–24^ because shorter B cell epitopes were chosen not to be presented by the major histocompatibility complex.^37^ In this study, the IL17A-peptide vaccine did not induce IL17A-autoreactive T cell activation (figure 4). These results indicate that the IL17A epitope-based vaccine safely exerts its therapeutic effects on IL17A-related pathologies in animal models.

The IL23/IL17 axis has been targeted as a pathogenic signalling pathway for psoriatic arthritis and SpA.^38^ Monoclonal antibodies targeting IL17A, such as secukinumab, ixekizumab and netakimab, have shown beneficial effects in clinical studies.^16–18 39 40^ From a basic standpoint, prophylactic IL17A blockade by monoclonal antibody effectively inhibits inflammation as well as bone destruction and formation in the axial and peripheral joints of HLA-B27 transgenic animals as experimental SpA models. Therapeutic IL17 inhibition with monoclonal antibodies also significantly mitigates SpA manifestations.^28^ Importantly, the therapeutic use of IL23 antibody does not alleviate the SpA phenotype in animal models,^41^ suggesting that IL17A is a suitable molecular target for the treatment of SpA as a therapeutic vaccine.

We also evaluated the therapeutic effect of IL17A in therapeutic MT-induced SpA model. The symptoms of SpA (arthritis score and joint thickness) was not improved in 20 days since onset of SpA symptoms. However, the symptoms tend to be mitigated, but not significant, in IL17A vaccine group, compared with KLH-control group (figure 5D and E). IL17A-specific antibody titre was higher at 7 weeks (49 days) since MT treatment, suggesting that the therapeutic effect of IL17A vaccine needs the certain level of antibody titre. Further longitudinal investigation need to be performed to evaluate the therapeutic effect of IL17A vaccine. In previous study, treatment with IL17A blockade with antibody has an therapeutic effect in MT-induced SpA model in HLA-B27 tg rats.^28^ SpA symptoms started to be improved after more than 10 days since IL17A antibody treatment. We speculated that the effect of therapeutic effect of IL17A vaccine may be active in much later compared with antibody therapy due to IL17A antibody induction time. These data led us to propose that combinatory treatment such as antibody therapy (fast acting) and vaccine therapy (long-term acting) might be more effective in SpA treatment.

In this study, our results showed that the IL17A peptide-based vaccine-induced IL17A-specific antibodies to effectively inhibit SpA manifestations in a rat SpA model. Therefore, IL17A antibodies induced via active immunisation may be additional therapeutic options for SpA treatment in the future.

## Study limitation

As a weak point of this study, we performed experiment with minimum number of animals (4–6 rats) in each group due to our experimental circumstances.

## Data Availability

All data related to this study for analysis are available upon reasonable request.
