# Supplementary material for: Peptide-based vaccine targeting IL17A attenuates experimental spondyloarthritis in HLA-B27 transgenic rats
Source: RMD Open. 2023 Feb 3;9(1):e002851. doi: 10.1136/rmdopen-2022-002851 (PMC9900070; doi:10.1136/rmdopen-2022-002851)
Supplement: Supplementary data [file rmdopen-2022-002851supp001.pdf]

Supplementary Figure 1

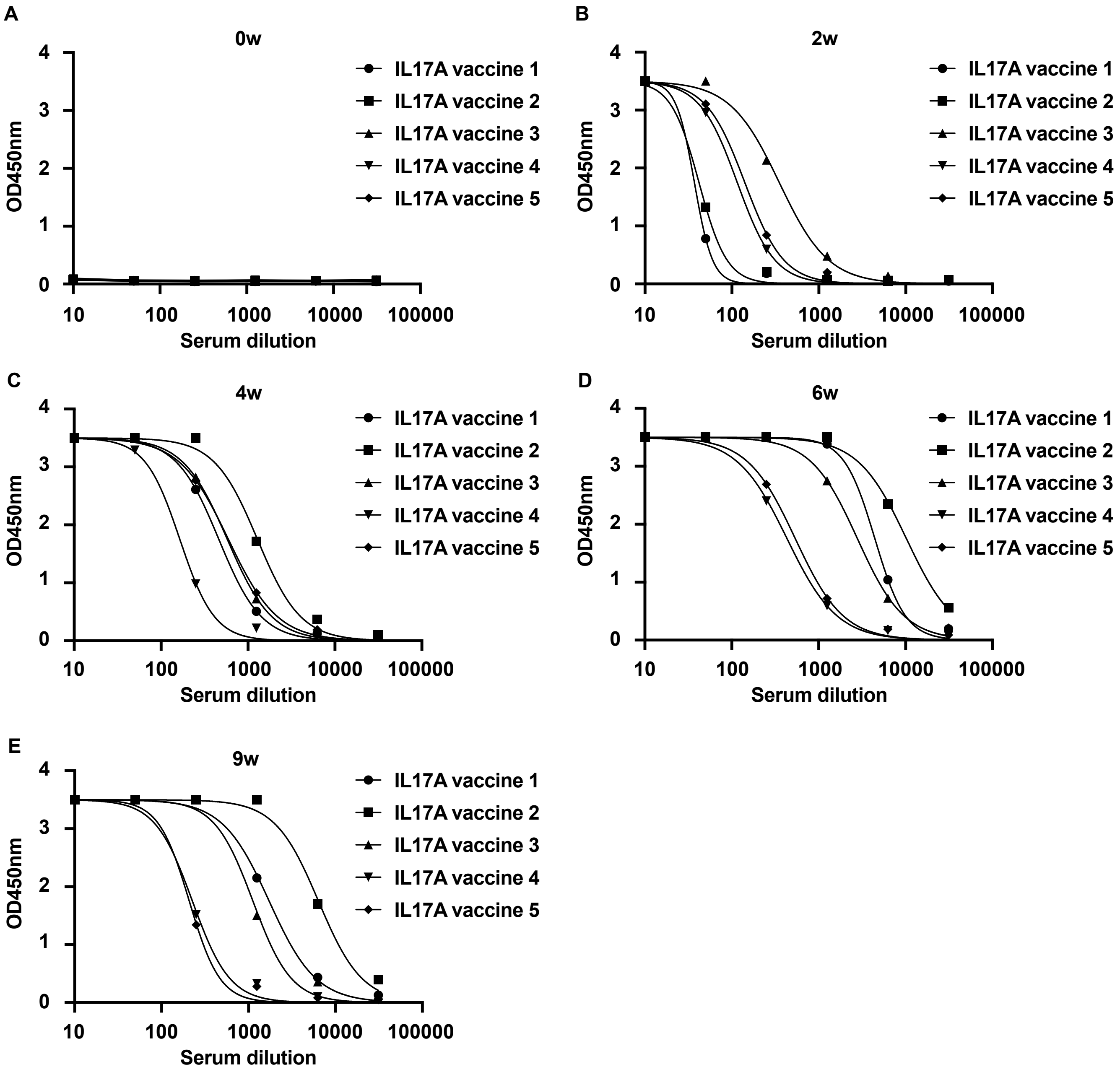

Supplementary Figure 1. IL17A epitope-specific antibody titer through 9 weeks. Antibody titers for IL17A epitope were measured by ELISA. Serum dilution from 10x to x31250. (**A**) 0w (Pre-vaccination), (**B**) 2w, (**C**) 4w, (**D**) 6w, (**E**) 9w, See also Figure 1.

Supplementary Figure 2

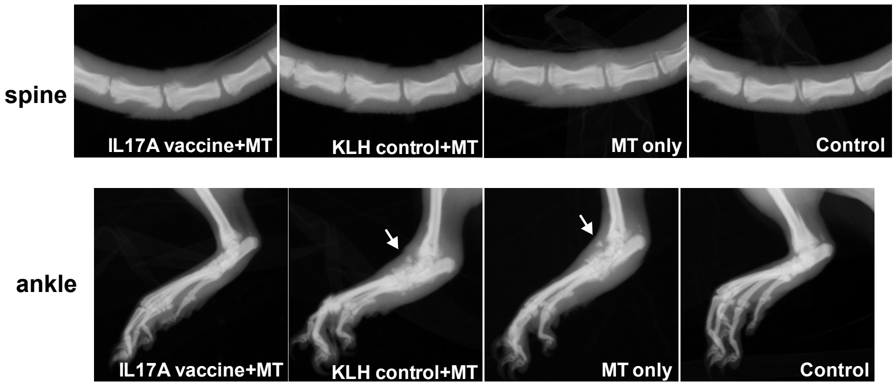

Supplementary Figure 2. IL17A vaccine suppresses enthesitis and bone remodeling in the joints of HLA-B27 transgenic rats. X-ray image of joint of the spine and ankle. Arrows indicate bone destruction. See also Figure 3.

Supplementary Figure 3

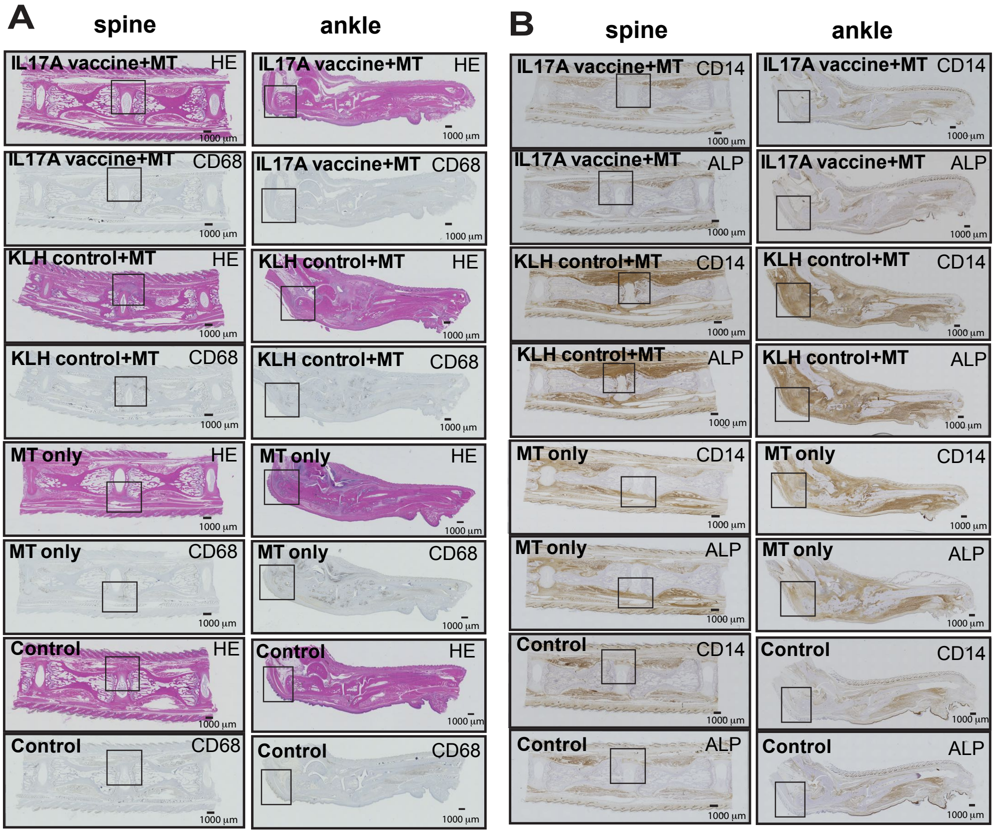

Supplementary Figure 3. IL17A vaccine suppresses enthesitis and bone remodeling in the joint of HLA-B27 transgenic rats. Representative whole images of spine and ankle with (**A**) HE and CD68 staining, and (**B**) CD14 and ALP staining. Enlarged image of black square (enthesis) is shown in Figure 3. See also Figure 3.

Supplementary Figure 4

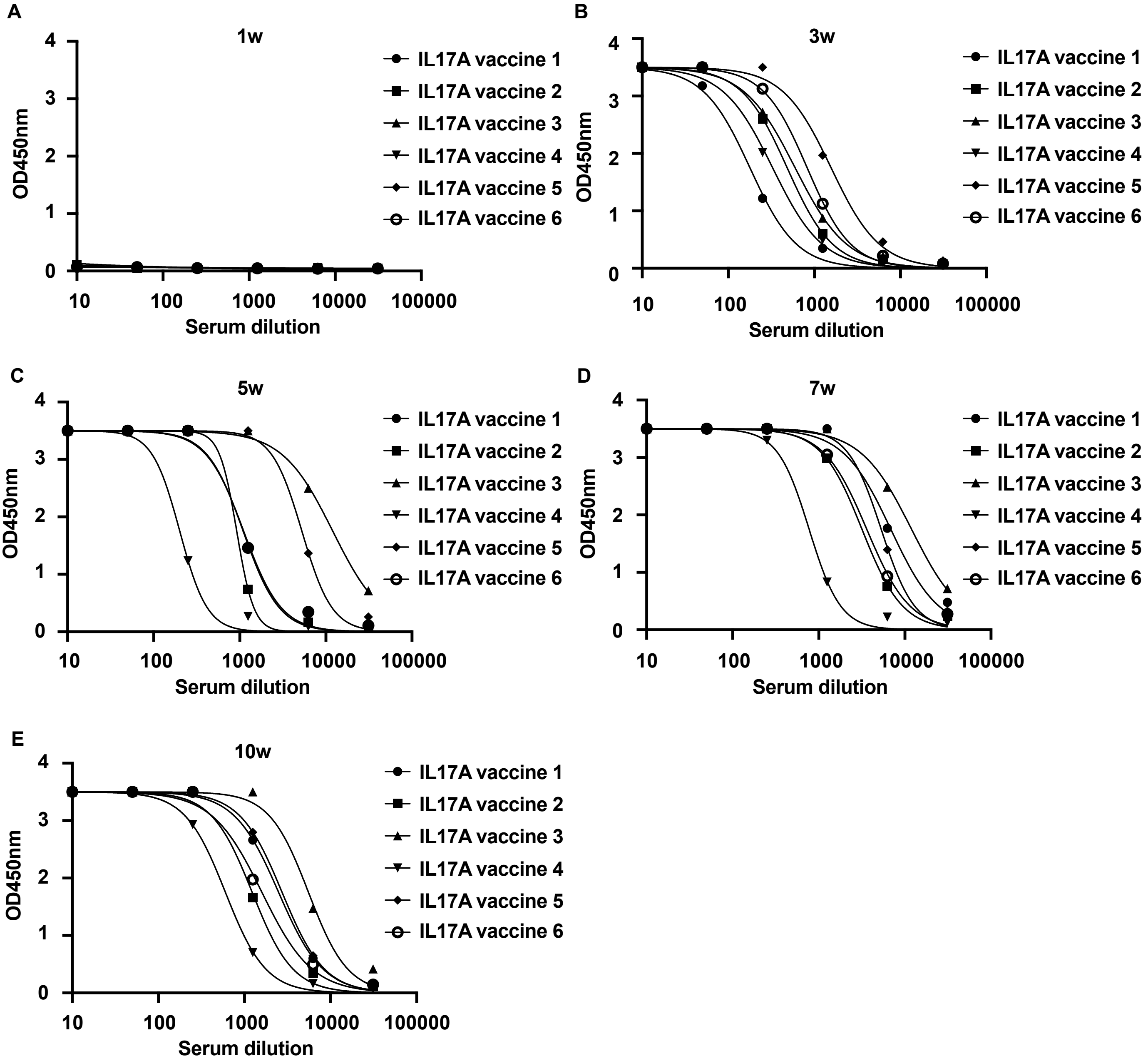

Supplementary Figure 4. IL17A epitope-specific antibody titer through 10 weeks in therapeutic model. Antibody titers for IL17A epitope were measured by ELISA. Serum dilution from 10x to x31250.
(**A**) 1w (Pre-vaccination), (**B**) 3w, (**C**) 5w, (**D**) 7w, (**E**) 10w, See also Figure 5.
